# Some Methods of Luminescence Efficiency Measurements*

**DOI:** 10.6028/jres.080A.039

**Published:** 1976-06-01

**Authors:** Alfred Bril, A. Willy de Jager-Veenis

**Affiliations:** Philips Research Laboratories, Eindhoven, The Netherlands

**Keywords:** Cathode-ray excitation, luminescence, luminescence standards, phosphors, quantum efficiencies, radiant efficiencies, UV excitation, x-ray excitation

## Abstract

Methods of absolute and relative radiant and quantum efficiency measurements are described for ultraviolet, visible, cathode-ray, and x-ray excitations. Data on some standard luminescent materials are given.

## 1. Introduction

Methods of absolute radiant and quantum efficiency measurements are given together with methods of relative efficiency measurements. The methods are especially suitable for powder materials for which the angular distribution of the emitted luminescent radiation is Lambertian.

The relative measurements are performed with the aid of standard phosphors, whose efficiencies have previously been determined by absolute measurements. Methods are given for excitation of the phosphors by ultraviolet and visible radiation, cathode rays and x rays.

For samples with non-Lambertian emission distributions, a method is described in which an Ulbricht’s sphere or an elliptical mirror is used.

## 2. Ultraviolet Excitation

**Figure 1:**
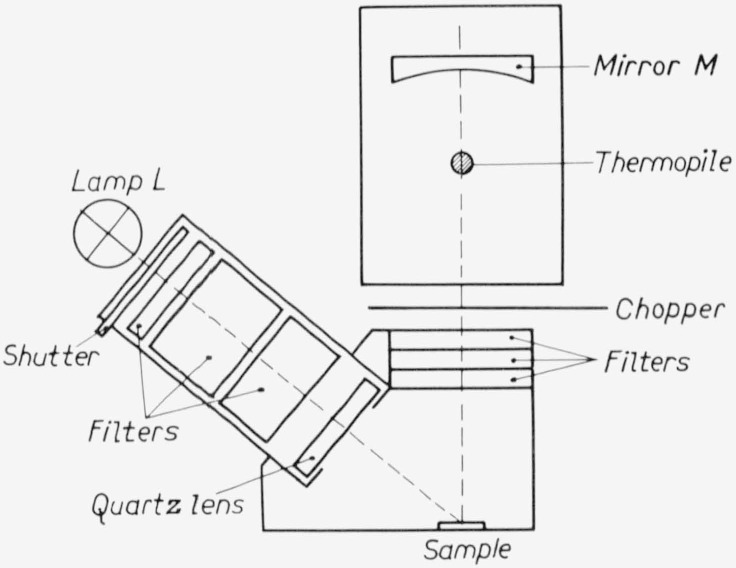
Schematic diagram of the apparatus for the efficiency measurements.

All powder phosphors are measured using a thick layer (thickness about 2 mm) at the irradiated side. The detection takes place perpendicular to the plane of the phosphor, the excitation is at an angle of 50° with that plane (see fig. 1). The excitation wavelength (*λ*_exc_) or regions are isolated from a high pressure mercury lamp by interference filters, the arc being focused on the phosphor with a quartz lens. In this way a high excitation density is reached, but generally well below the excitation region where saturation effects start. This is especially advantageous when a relatively insensitive thermoelement is used as a detector.

The radiant efficiencies, from which the quantum efficiencies are calculated, are determined directly (when the spectral power distribution is known).

### 2.1. Relative Measurements, Giving Absolute Efficiency Values

Phosphors can be measured with respect to the following standard samples whose efficiency is generally agreed upon.

- The standards issued by the National Bureau of Standards, Washington, D.C. (See ref. [1–4]).
- Sodium salicylate (See ref. [5]).
  This phosphor is also suitable for excitation in the far ultraviolet (vacuum ultraviolet) because of its constant efficiency as a function of λ_exc_ up to 350 nm.
- The standard “Ekta S10”^1^ proposed by Grum [6].
- “Lumogen T red GG,” which can be used in the excitation region between 190 and 550 nm [7].

### 2.2. Absolute Measurements

The absolute radiant efficiency can in fact be determined with the aid of a relative measurement, being the ratio of the amount of emitted power and that of the absorbed exciting power [1].^2^ For one or two wavelengths the absolute efficiencies can be determined. For other λ_exc_ the *relative* excitation spectrum can be determined from which the absolute efficiency at any λ_exc_ can be derived.

For this determination three quantities are measured:

- The diffuse reflection of the exciting radiation against BaSO_4_ for which the reflection is known.
- The luminescence + reflection of the exciting radiation (without using a filter).
- The luminescence of the phosphor, using a filter between phosphor and detector that passes only the luminescence.
  From these three measured quantities the reflection and radiant efficiency of the phosphor can be determined.

The expressions found for the radiant efficiency *η_p_* and the reflection *r_p_* are given here for the case of using as a detector a thermopile or thermoelement with flat radiant response. Three emf’s are measured, viz, *V_R_* due to the reflection standard (e.g., BaSO_4_ [8], reflection *R*), *V_P_* due to the phosphor (luminescence intensity *L* + reflected exciting radiation of intensity *I*) and *V_P_*, *_F_* due to the phosphor when a filter *F* absorbing the exciting radiation is placed in front of the detector. We assume that the filter has a transmission *τ* in the emission region of the phosphor. This leads to the following equations:

$$\begin{array}{l} \;CV_{R}=IR\\ \;CV_{P}=Ir_{p}+L\\ CV_{P,F}=\tau L \end{array}$$

where *C* is a constant.

After solving for *r_p_* and *L* we find

ηp=LI(1−rp)=Rτ(1−rp)VP,FVRrp=RVP−VP,F/τVR

As a cross-check the reflection found in this way can be compared with that measured directly with a spectrophotometer.

The method described can be used in the same way for the case of a varying spectral response of the detector and/or a varying spectral transmission of the filter, even when the filter transmits partly in the region of the exciting radiation. Of course the equations become somewhat more complicated in this case.

The quantum efficiency *q_p_* is found from the radiant efficiency by

$$q_{p}=\eta_{p}\frac{\int \lambda_{p}(\lambda )d\lambda }{\lambda_{\mathrm{exc}}\int p(\lambda )d\lambda }$$

where *p*(*λ*) is the emitted luminescent power and *λ*_exc_ is the exciting wavelength.

The NBS standards mentioned in 2.1 are not excited in the visible (only No. 1030 would be suitable in the blue-violet) region. Therefore, other standards are necessary in the visible region. These can be found among the “lumogen” phosphors. A yellow luminescent lumogen was described by Kristianpoller and Dutton [9], yellow and red ones by Vavilov [10]. Morgenshtern, Neustruev and Epshtein [11] and Küttner, Selzle and Schlag [12]. The latter used 5-(p-dimethylaminobenzyliden)-barbituric acid as a red lumogen; they found a quantum efficiency of 45 percent at *λ*_exc_^=^ 405 nm.

**Figure 2:**
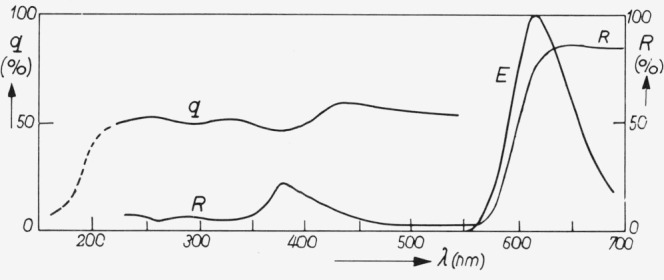
“Lumogen T red GG” *q* = quantum efficiency, *R* = diffuse reflection and *E* = spectral power distribution.

We chose the red luminescent “Lumogen T red GG” which was already mentioned in section 2.1. It is commercially available from the Badische Anilin und Soda Fabrik (Ludwigshafen, Germany). The properties of the phosphor are described in reference [7]. It has a red luminescence and shows a quantum efficiency which is not quite constant but varies in a limited range between 40 percent and 60 percent in the spectral region between 220 nm and 550 nm (see fig. 2).

**Figure 3:**
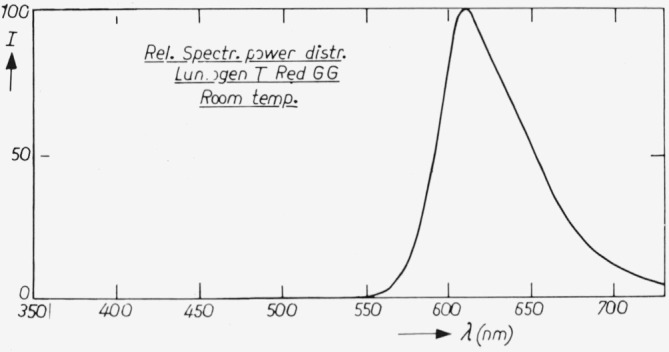
Spectral power distribution of “Lumogen T red GG” at room temperature. *I* denotes the spectral radiant power in arbitrary units.

**Figure 4:**
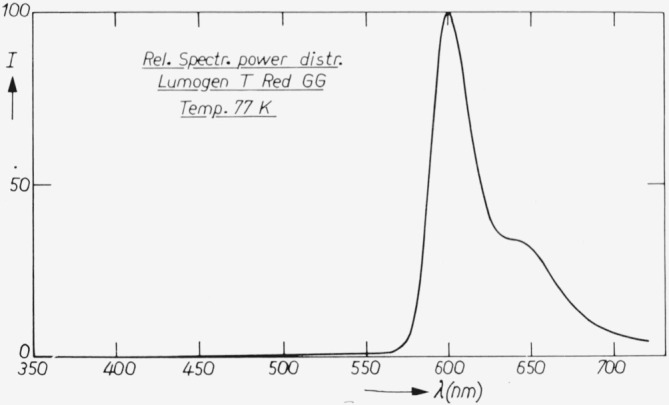
Spectral power distribution of “Lumogen T red GG” at liquid nitrogen temperature. See further subscript fig. 3.

**Figure 5:**
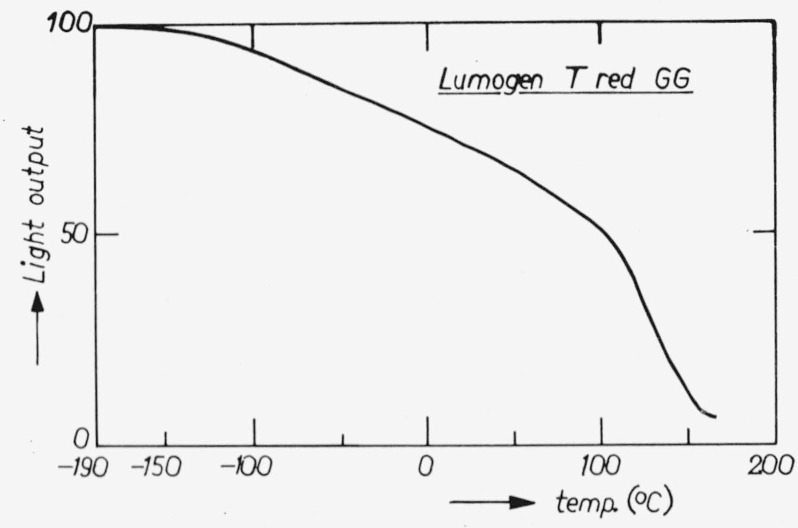
Temperature dependence of the luminescence of “Lumogen T red GG” for *λ*_exc_ = 365 nm.

The spectral power distributions at room temperature and liquid nitrogen temperature are given in figures 3 and 4. The temperature dependence curve is given in figure 5 for *λ*_exc_ = 365 nm. The quantum efficiency together with the diffuse spectral reflection are given in figure 2. An important advantage of this phosphor over liquid standards like rhodamine B is that the absorption is high in the whole region, the lowest value being 78 percent near *λ* = 380 nm (diffuse reflection ≈ 22 percent).

**Figure 6:**
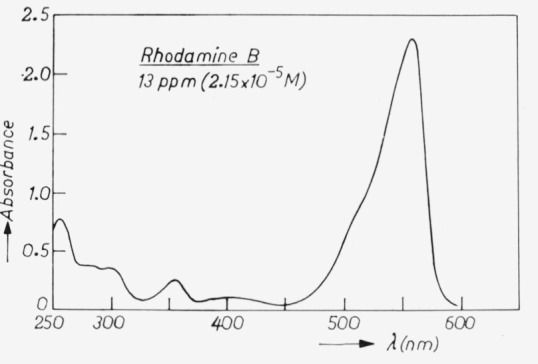
Spectral absorbance of rhodamine B.

The absorption of rhodamine B is given in figure 6, showing the enormous variation through the spectrum leading to a similar large variation in light output. Another drawback of liquid samples is the different geometry of the set-up needed for the measurement.

Various authors have reported measurements using 254 nm mercury vapour discharge excitation. Here we give additional measurements on some standards for longer wavelength excitation at λ_exc_ = 365 nm. The phosphors measured were sodium salicylate, the “Ekta S10” sample, introduced by Grum [6] and “Lumogen T red GG” [7] (see tables 1 and 2).

The results for diffuse reflection at the exciting and emission wavelength, the practical and intrinsic radiant efficiencies, and the practical and intrinsic quantum efficiencies are given.

Because of the thick layer used, a correction has to be made for the loss of the light absorbed in the layer. The intrinsic radiant efficiency *η_i_* can then he approximated by [2]

$$\eta_{i}=\frac{2}{1+r_{\infty }}\eta_{p}$$

where *r*_∞_ is the reflection coefficient of the phosphor for an infinitely thick layer.

**Figure 7:**
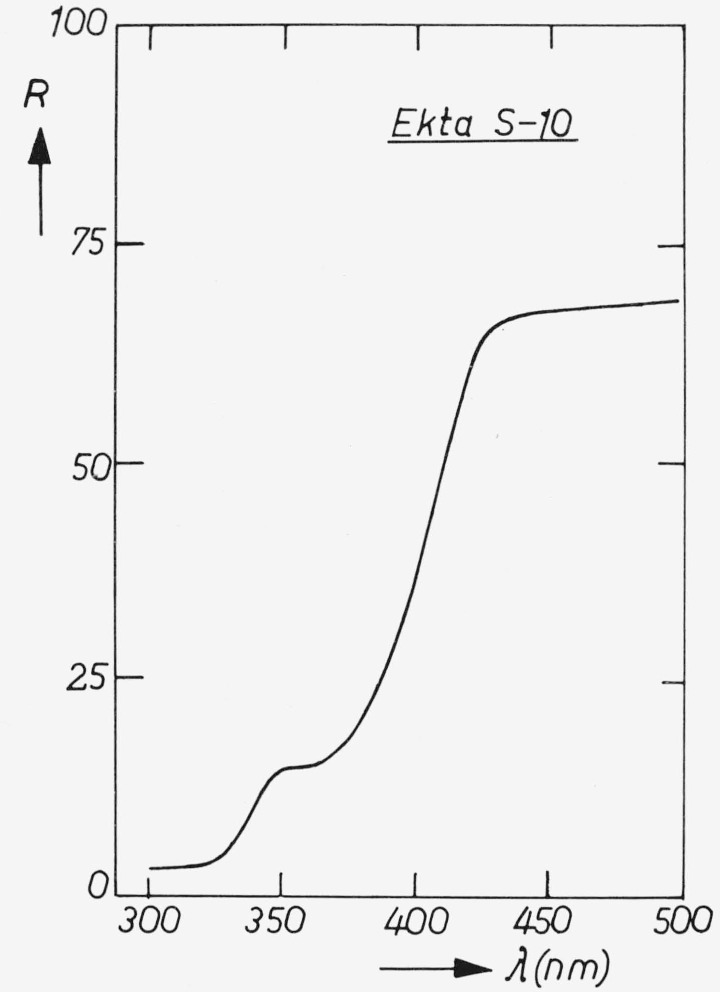
Diffuse reflection of “Ekta S10.”

**Figure 8:**
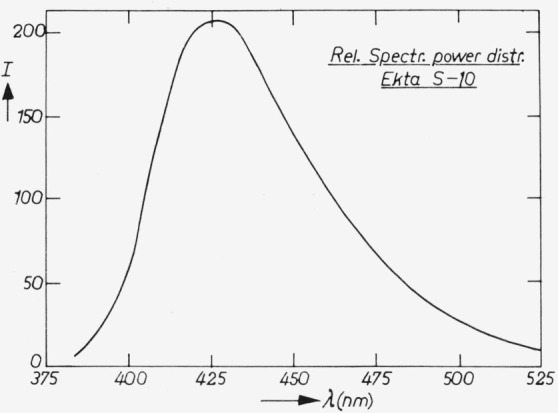
Spectral power distribution of “Ekta S10.” See further subscript fig. 3.

The diffuse reflection of “Ekta S10” is given in figure 7, the spectral power distribution in figure 8.

The efficiency data for Na-salicylate at λ_exc_ = 260 nm can be compared with the data given in Samson’s book (ref. [3]) which are discussed by us in reference [7], together with some additional data.

Polarization effects in our measurements proved to be negligible, as may be expected for powder materials. Measurements were carried out with incident polarized UV radiation, in two directions perpendicular to each other.

The stability of the lumogen was also tested as well as the dependence on excitation density. During one month the efficiency of the lumogen was measured every two days. The stability in time proved to be very good; no changes were observed within the error of measurement, which was of the order of ±10 percent.

The efficiency values were not affected even when the intensity of the UV-radiation was attenuated a thousand times.

## 3. Excitation in Selected Narrow Absorption Peaks

A method to determine the efficiencies of phosphors that have a small absorption of a few percent in narrow, well defined excitation levels (for the normal case of *λ*_exc_ ≤ *λ*_em_) was described earlier by us [13, 14]. Examples of these powders are rare-earth activated phosphors, such as YVO_4_ – Eu^3+^ and NaYF_4_ – Er^3+^, where the (visible) excitation peaks are those of the rare-earth ion. The host lattice absorbs in the UV region.

**Figure 9:**
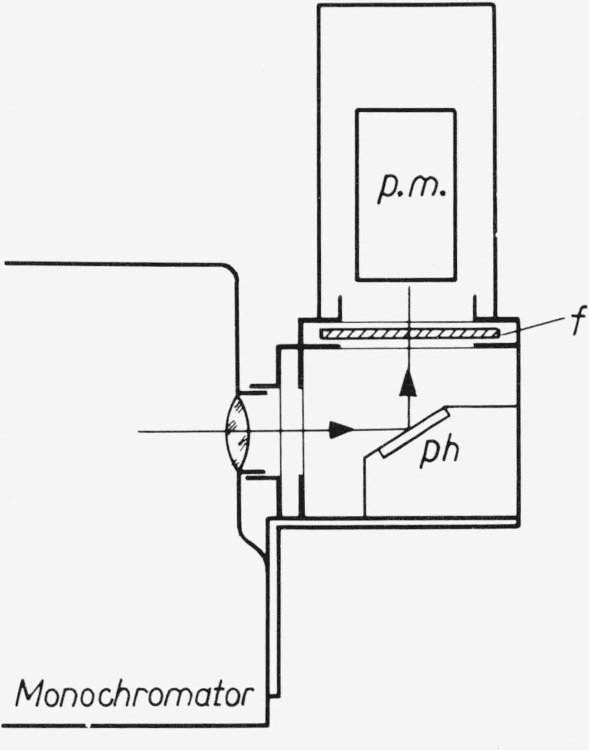
Schematic diagram of the experimental set-up: ph = phosphor sample, f = filters, pm = photomultiplier.

A diagram of the set-up is shown in figure 9. The phosphor is irradiated via a scanning monochromator. Two measurements have to be carried out, differing only in the filter used in front of the photomultiplier.

**Figure 10:**
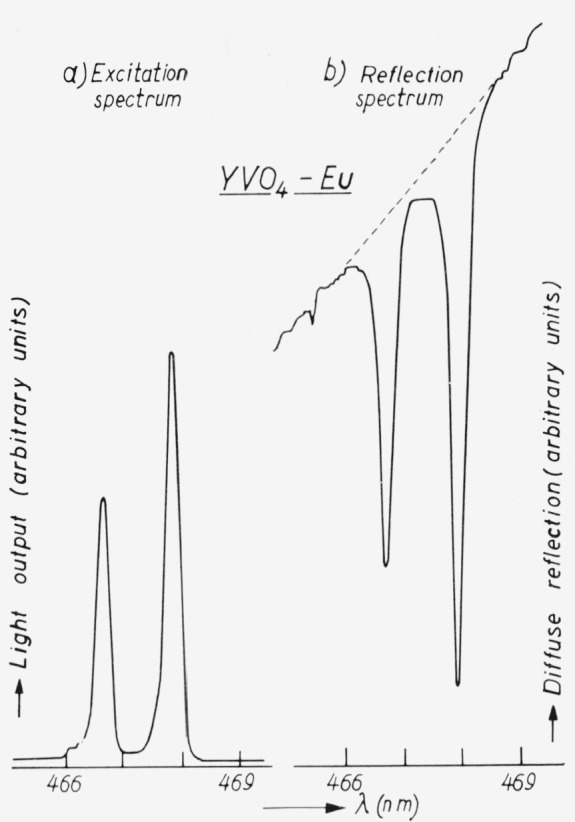
Relative light output of Eu^3+^-emission (curve a) and diffuse reflection (curve b) as a function of wavelength for YVO_4_-*Eu*. For curve b the zero line is suppressed (the absorption peak has a depth of about 13 percent).

One filter transmits only the light reflected from the sample, giving the absorption spectrum. In the second measurement the other filter selects the emission wavelength region, thus obtaining the excitation spectrum of that emission. The curves are of the type shown in figure 10 for YVO_4_ – Eu^3^+. The efficiency is calculated as follows.

The radiant efficiency is the ratio of the emitted power *E* to the absorbed exciting power *A.* The latter is determined by the area under the absorption curve of a certain peak with correction for the transmission *τ_A_* of the filter used and for the photomultiplier response *G*(*λ_A_*) in the absorption region. The emitted power *E* is determined by the corresponding area under the excitation curve with correction for the transmission *τ_E_* of the filter used in this case and the response *G*(*λ_E_*) in the emission region. For single narrow peaks we can take the ratio of the ordinate maxima in the absorption and excitation spectra *U_A_* and *U_E_* respectively, instead of the area. We then find for the emitted power

$$E=U_{E}\frac{\int p(\lambda_{E})d\lambda_{E}}{\int p(\lambda_{E})G(\lambda_{E})\tau_{E}(\lambda_{E})d\lambda_{E}}$$

where *p*(*λ_E_*)*dλ_E_* is the relative emitted power in a region *dλ_E_* (integration extended over the total spectral region of the emission). The absorbed power is given by

$$A=\frac{U_{A}}{G(\lambda_{A})\tau_{A}}.$$

Then the radiant efficiency *η* is

$$\eta =\frac{E}{A}=\frac{U_{E}\tau_{A}G(\lambda_{A})\int p(\lambda_{E})d\lambda_{E}}{U_{A}\int p(\lambda_{E})G(\lambda_{E})\tau_{E}(\lambda_{E})d\lambda_{E}}.$$

The quantum efficiency *q* is derived from the radiant efficiency by

$$q=\eta \frac{\int \lambda_{EP}(\lambda_{E})d\lambda_{E}}{\lambda_{A}\int p(\lambda_{E})d\lambda_{E}}.$$

The error in this type of measurement may be of the order of 10–25 percent, depending on the value of the absorption. This large error is caused by the low value of the absorption in the rare-earth ion.

## 4. Cathode-Ray Excitation

The radiant efficiency *η_p_* for cathode-ray excitation [2–4, 15, 16] is generally defined as the ratio of the amount of emitted luminescent power in the spectral region under consideration to the power of the incident cathode-ray beam (and not to the power *absorbed* by the phosphor layer). Thus no correction is made for the loss due to reflection of primary electrons [2, 15, 16].

**Figure 11:**
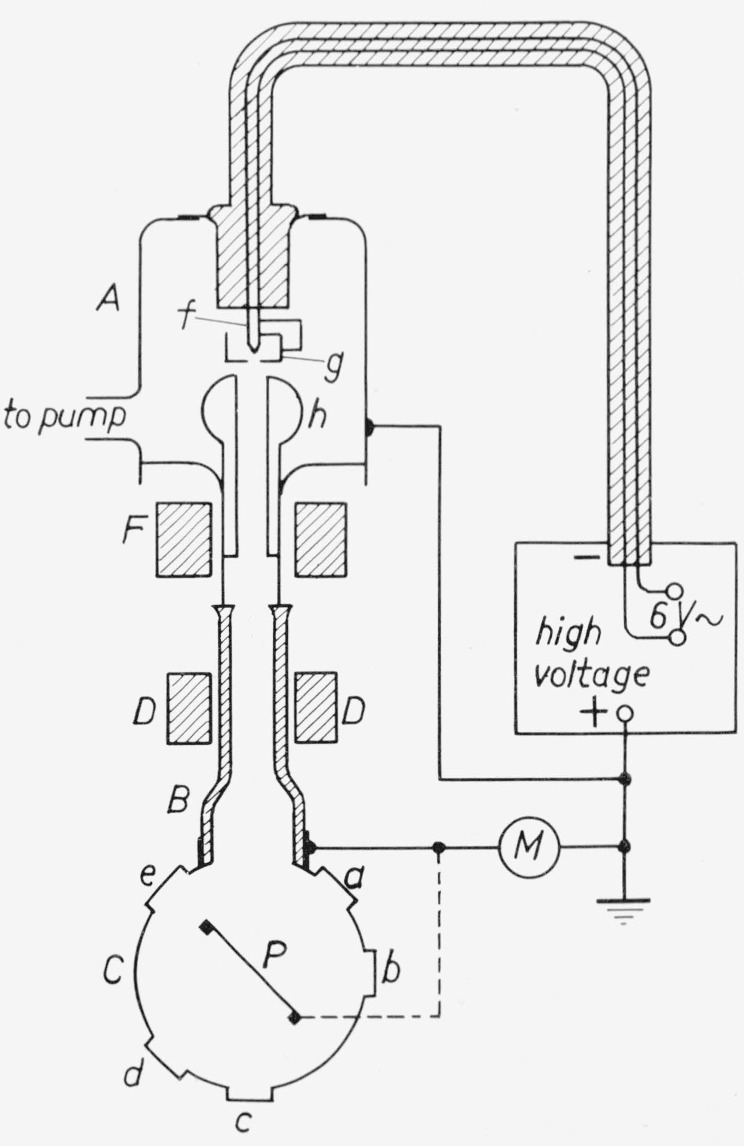
Experimental set-up for efficiency measurements with C. R. excitation. A. metal chamber, h. anode, f. filament, g. cap, F. focusing coil, D. deflection coils, B. glass tube, C. metal cylinder, P. target plate, a, b, c, and d quartz windows, M. microammeter.

In this case two really absolute measurements are necessary, viz., that of the emitted power and that of the power of the cathode-ray beam (fig. 11).

The measurements are carried out on thick layers at the irradiated side. Precautions should be taken to ensure that charging up of the layer is negligible.

The radiant output of the phosphor was compared with the radiation of a standard lamp which was diffusely reflected by a MgO layer. A thermopile was used [2]|as a detector.

## 5. X-Ray Excitation

**Figure 12:**
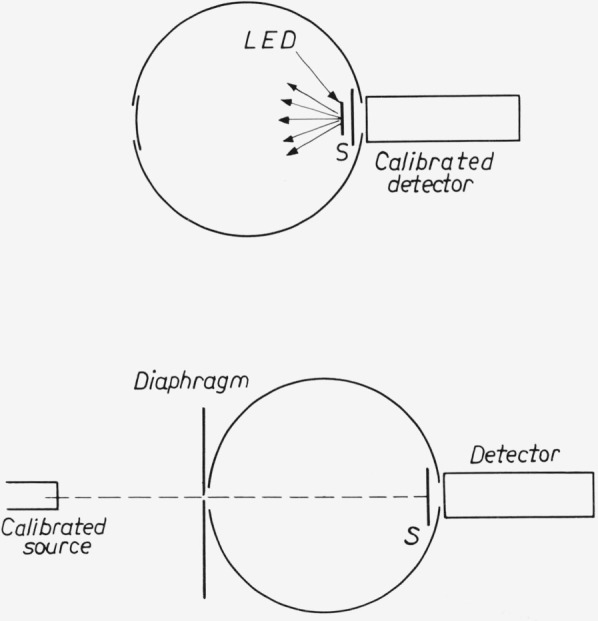
Diagram for efficiency measurement of light emitting diodes.

To measure radiant efficiencies with x-ray excitation [17–19] *thin* phosphor layers are used (≈ 100*μ*m). This is necessary to minimize the loss in light output due to scattering and absorption of the emitted luminescence. The total back-screen emission is collected by a 2*π*-geometry elliptical mirror and focused onto the photomultiplier detector (see fig. 12), which is calibrated in absolute units (A/W). The x-ray absorption coefficients are measured, with a scintillation crystal as well as calculated from the tables of Storm and Israel [20].

## 6. Measurements of Light-Emitting Diodes, Crystals, etc

In cases where the angular distribution of the emitted radiation does not obey Lambert’s law it is not sufficient to measure the emitted radiation in one direction but the total radiation should be determined. This can be carried out with the aid of an Ulbricht’s sphere or with an elliptical mirror.

**Figure 13:**
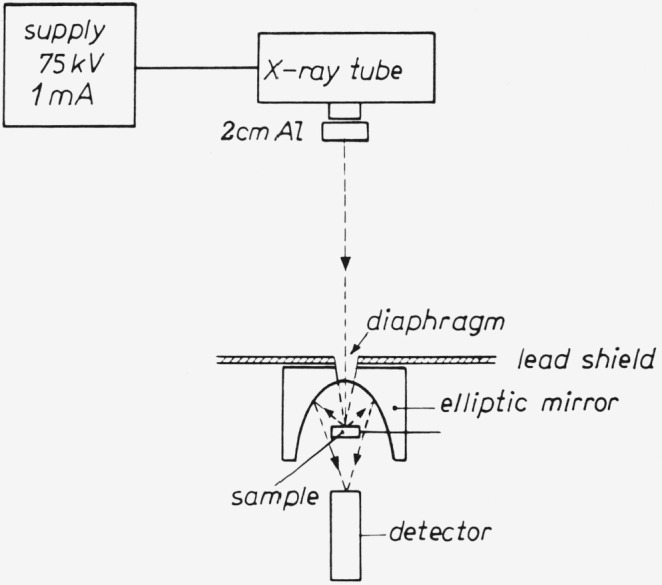
Experimental set-up for x-ray efficiency measurements.

The absolute output can be calibrated in two ways (see fig. 13):

1. the luminescent output is measured with a calibrated detector (A/W · cm^2^), e.g., a 150 CV or 150 UV photocell (calibration National Physical Laboratory, Teddington, England).
2. the luminescent output is compared with the output of a calibrated standard lamp, e.g., a 200 W or 1000 W tungsten - halogen lamp; calibrated by the National Bureau of Standards in Washington, D.C. (W/nm · cm^2^). In this case the diode to be measured is replaced by a BaSO_4_-coated screen S.

The use of a 2 *π*-geometry elliptical mirror [21] instead of an Ulbricht’s sphere gave nearly the same results.

## Figures and Tables

**Table 1:** Efficiencies at *λ*_exc_ = 365 nm

Phosphor	Diffuse reflection at		Radiant efficiency		Quantum efficiency	
	*λ* = 365 nm %	*λ*_em_ %	*η_p_* %	*η_i_* %	*q_p_* %	*q_i_* %
						
Na-salicylate	30	80	33	37	37	41
“Ekta S10”	15	67	37	45	41	50
“Lumogen T red GG”	13		25		41	

**Table 2:** Efficiencies at *λ*_exc_ = 260 nm

Phosphor	Diffuse reflection at		Radiant efficiency		Quantum efficiency	
	λ = 260 nm %	*λ*_em_ %	*η_p_* %	*η_i_* %	*q_p_* %	*q_i_* %
						
Na-salicylate	7	80	34	37	55	60
“Lumogen T red GG”	6		22		53	

## Footnotes

1 In order to describe materials and experimental procedures adequately, it is occasionally necessary to identify commercial products by manufacturer’s name or label. In no instance does such identification imply endorsement by the National Bureau of Standards, nor does it imply that the particular product or equipment is necessarily the best available for that purpose.

2 Figures in brackets indicate the literature references at the end of this paper.
